# Unexpected Activity of a Novel Kunitz-type Inhibitor: INHIBITION OF CYSTEINE PROTEASES BUT NOT SERINE PROTEASES *[Fn FN2]

**DOI:** 10.1074/jbc.M116.724344

**Published:** 2016-07-15

**Authors:** David Smith, Irina G. Tikhonova, Heather L. Jewhurst, Orla C. Drysdale, Jan Dvořák, Mark W. Robinson, Krystyna Cwiklinski, John P. Dalton

**Affiliations:** From the ‡ School of Biological Sciences and; § School of Pharmacy, Medical Biology Centre, Queen's University Belfast, Belfast BT9 7BL, Northern Ireland, United Kingdom

**Keywords:** cysteine protease, enzyme inhibitor, enzyme kinetics, host-pathogen interaction, molecular modeling, parasite, protease inhibitor, protein-protein interaction, serine protease

## Abstract

Kunitz-type (KT) protease inhibitors are low molecular weight proteins classically defined as serine protease inhibitors. We identified a novel secreted KT inhibitor associated with the gut and parenchymal tissues of the infective juvenile stage of *Fasciola hepatica,* a helminth parasite of medical and veterinary importance. Unexpectedly, recombinant KT inhibitor (rFhKT1) exhibited no inhibitory activity toward serine proteases but was a potent inhibitor of the major secreted cathepsin L cysteine proteases of *F. hepatica*, FhCL1 and FhCL2, and of human cathepsins L and K (*K_i_* = 0.4-27 nm). FhKT1 prevented the auto-catalytic activation of FhCL1 and FhCL2 and formed stable complexes with the mature enzymes. Pulldown experiments from adult parasite culture medium showed that rFhKT1 interacts specifically with native secreted FhCL1, FhCL2, and FhCL5. Substitution of the unusual P1 Leu^15^ within the exposed reactive loop of FhKT1 for the more commonly found Arg (FhKT1Leu^15^/Arg^15^) had modest adverse effects on the cysteine protease inhibition but conferred potent activity against the serine protease trypsin (*K_i_* = 1.5 nm). Computational docking and sequence analysis provided hypotheses for the exclusive binding of FhKT1 to cysteine proteases, the importance of the Leu^15^ in anchoring the inhibitor into the S2 active site pocket, and the inhibitor's selectivity toward FhCL1, FhCL2, and human cathepsins L and K. FhKT1 represents a novel evolutionary adaptation of KT protease inhibitors by *F. hepatica*, with its prime purpose likely in the regulation of the major parasite-secreted proteases and/or cathepsin L-like proteases of its host.

## Introduction

*Fasciola hepatica* is a zoonotic parasitic helminth common in temperate and sub-tropical regions of the world. The parasite is responsible for causing the disease fasciolosis in hundreds of millions of livestock, principally sheep and cattle. This results in reduced feed conversion, decreased dairy production, inferior meat quality and parasite-related mortality, thus costing the agricultural industry an estimated United States $3 billion annually (1, 2). *F. hepatica* is also estimated to infect up to 17 million people throughout the world, primarily in developing countries, with ∼180 million at risk of infection (3, 4). The mammalian host becomes infected following ingestion of grass or other vegetation contaminated with *F. hepatica* cysts (metacercariae). The parasites then exocyst in the host duodenum and penetrate through the intestinal wall before migrating to the liver and bile ducts. To facilitate this journey, the parasite excretes and secretes an array of molecules that come into contact with host cells and tissues, the most abundant being proteases and protease inhibitors (5, 6). These molecules are important for the parasite's survival within its host and perform roles in immunomodulation, immune evasion, feeding, parasite development, and protein regulation (5–9).

Transcriptomic data analysis of the infective newly excysted juvenile (NEJ) ^5^ stage of *F. hepatica* identified a cDNA sequence that encodes a protein with homology to a Kunitz-type (KT) serine protease inhibitor (10). In their monomeric form, KT protease inhibitors are typically low molecular mass proteins of 6–8 kDa. They contain six cysteine residues that form three conserved disulfide bonds in a 1–6, 2–4, and 3–5 arrangement that maintains structural integrity of the inhibitor and allows presentation of a protease-binding loop at its surface (see Fig. 2) (11–13). A highly exposed P1 active site residue at position 15, which inserts into the S1 site of the cognate protease, is located at the peak of the binding loop and is of prime importance in determining the specificity of serine protease inhibition (14). The P1 site residue is usually arginine (Arg) or lysine (Lys), both of which have a positively charged side chain (11) and are the preferential site of interaction for the digestive protease trypsin; thus, KT protease inhibitors are classically associated with trypsin inhibition (*e.g.* bovine pancreatic trypsin inhibitor, BPTI) (15–17). Other serine proteases often inhibited by KT inhibitors include the digestive enzyme chymotrypsin, neutrophil elastase, and several serine proteases involved in the blood coagulation cascade, such as thrombin, kallikrein, and various other tissue factors (12, 17–21). The P1 residue in the *F. hepatica* KT is a leucine (Leu), which has been found in certain KT inhibitors that have a greater specificity for chymotrypsin over trypsin (11).

Although genes encoding KT protease inhibitors have been identified in a number of parasitic organisms (11), only a few have been characterized functionally. However, because of their putative protease-inhibition properties, they are proposed to be important in parasite defense (11). For example, in intestinal nematodes such as hookworms (22–24) and cestodes such as *Echinococcus granulosus* (25, 26), KT protease inhibitors may protect the parasite by inhibiting potentially harmful host digestive enzymes. However, in the guts of blood-feeding schistosome parasites (27, 28) and the secretions of biting insects (29) and ticks (30–32), KT inhibitors are suggested to obstruct blood coagulation enzymes to prevent blood clotting. A KT inhibitor with trypsin inhibitory activity has previously been identified in soluble extracts of adult *F. hepatica* taken from the bile ducts of ruminants (33), but its function remains obscure as it exhibited no activity against the major coagulation enzymes and only low activity (*K_i_* = 143 nm) against trypsin.

Here, we report the discovery of a KT protease inhibitor (FhKT1) from the infective and tissue invasive stage of *F. hepatica* that exhibits no inhibitory activity against serine proteases but has potent activity against cathepsin L-like cysteine proteases. Substitution of the atypical P1 Leu^15^ that projects from the exposed reactive loop of the inhibitor with an Arg residue modestly reduces its cysteine protease inhibition profile but endows the protein with trypsin-inhibitory activity. Molecular modeling of the interaction of FhKT1 with the major secreted parasite cathepsin L cysteine proteases and human cathepsin L-like cysteine proteases indicated the importance of the P1 Leu^15^ in docking the inhibitor into the S2 active site of these enzymes and suggested key inhibitor-enzyme interactions that impart FhKT1 its unique inhibitory properties.

## Results

### 

#### 

##### Expression of fhkt1 in Newly Excysted Juveniles in F. hepatica

**FIGURE 1.**
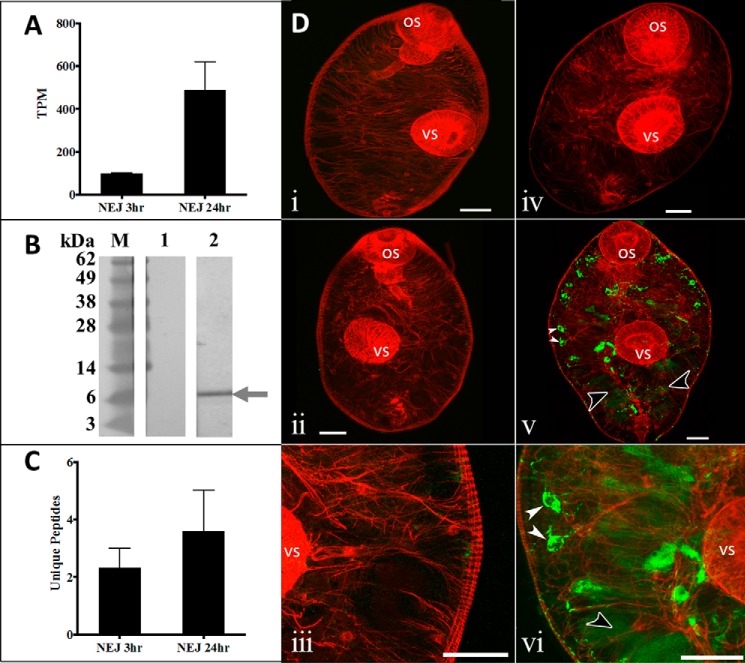
**FhKT1 is a temporally regulated and secreted protein in *F. hepatica* NEJ.** *A,* graphical representation of *fhkt1* expression within NEJs 3 and 24 h post-excystment in transcripts per kilobase million (*TPM*). *Error bars* indicate standard deviation of two separate experiments. *B,* Western blot analysis of RPMI 1640 culture medium from *F. hepatica* NEJs cultured for 24 h. Following the transfer of protein from LDS-polyacrylamide gel onto a nitrocellulose membrane by Western blotting, blots were spliced and probed with pre-immune mouse antiserum (*lane 1*) and anti-FhKT1 monoclonal antibodies raised in mice (*lane 2*). The immunoreactive band in *lane 2* corresponding to FhKT1 is indicated by a *gray arrow. M,* molecular mass marker. *C,* graphical representation of mass spectrometry analysis of FhKT1 present in RPMI 1640 culture medium from *F. hepatica* NEJs cultured for 3 and 24 h, shown as unique peptide number. *Error bars* indicate standard deviation of three separate experiments. *D,* immunolocalization of FhKT1 in NEJs by confocal scanning laser microscopy. NEJs were fixed and stained as described under “Experimental Procedures.” FhKT1 expression in NEJs from two time points were compared, 3 h post-excystment (*panels i–iii*) and 24 h post-excystment (*panels iv–vi*). Fixed NEJs were probed with either anti-FhKT1 polyclonal antibodies raised in rabbit (*panels ii, iii, v,* and *vi*) or rabbit pre-immune antiserum (negative controls, *panels i* and *iv*), followed by the secondary antibody, fluorescein isothiocyanate (FITC)-labeled goat anti-rabbit IgG. No FITC staining was observed in the negative controls (*panels i* and *v*). Very little FITC staining (*green fluorescence*) was evident in the 3-h NEJs (*panels ii* and *iii*). FITC staining was observed in 24-h NEJs (*panels v* and *vi*), with FhKT1 being evident in the parenchyma (particularly concentrated in parenchymal cell bodies, *white arrowheads*) and gut (*black arrowheads*). *Panels iii* and *vi* and high power images of *panels ii* and *v*, respectively. All specimens were counter-stained with phalloidin-tetramethylrhodamine isothiocyanate (TRITC) to stain muscle tissue (*red fluorescence*) and provide structure. *OS,* oral sucker; *VS,* ventral sucker. *Scale bars,* 20 μm. *M,* molecular mass markers.

Transcriptome analysis of the newly excysted juvenile stage showed that *fhkt1* is expressed by the early *F. hepatica* stages that invade the host. Differential gene expression analysis during the first 24 h post-excystment shows that *fhkt1* gene is up-regulated in NEJs 24 h post-excystment compared with NEJs 3 h post-excystment (Fig. 1*A*). Western blot analysis using anti-FhKT1 antibodies revealed an ∼7-kDa band in medium in which NEJs were cultured for 24 h (Fig. 1*B*, *lane 2*), confirming that FhKT1 is secreted by this parasite the 1st day after excystment. Mass spectrometry analysis of NEJ-secreted products revealed that FhKT1 is released into medium by both 3- and 24-h post-excystment NEJs (Fig. 1*C* and supplemental file 1).

To localize FhKT1 expression within the NEJ, confocal microscopy was carried out on 3- and 24-h post-excystment NEJs. These were probed with polyclonal anti-FhKT1 antibodies and counter-stained with TRITC-labeled phalloidin to provide NEJ structural visualization of muscle tissue under fluorescence microscopy. Little fluorescence was observed in NEJs 3 h post-excystment using anti-FhKT1 (Fig. 1*D*, *panels ii* and *iii*). By contrast, in NEJs 24 h post-excystment, bright fluorescence was observed in the parenchymal tissue and particularly concentrated in parenchymal cell bodies (*white arrowheads*) (Fig. 1*D*, *panels v* and *vi*). Diffuse staining was observed within the NEJ gut (*black arrowheads*) (Fig. 1*D*, *panels v* and *vi*). This increase in fluorescence in 24-h NEJs compared with 3-h NEJs is consistent with the up-regulated transcript and protein expression observed in 24-h NEJs (Fig. 1, *A* and *C*). No FITC fluorescence was observed in negative control NEJs (3 and 24 h post-excystment) probed with pre-immune sera (Fig. 1*D*, *panels i* and *iv*).

##### F. hepatica Inhibitor FhKT1 Exhibits a Unique Specificity for Cysteine Proteases

**FIGURE 2.**
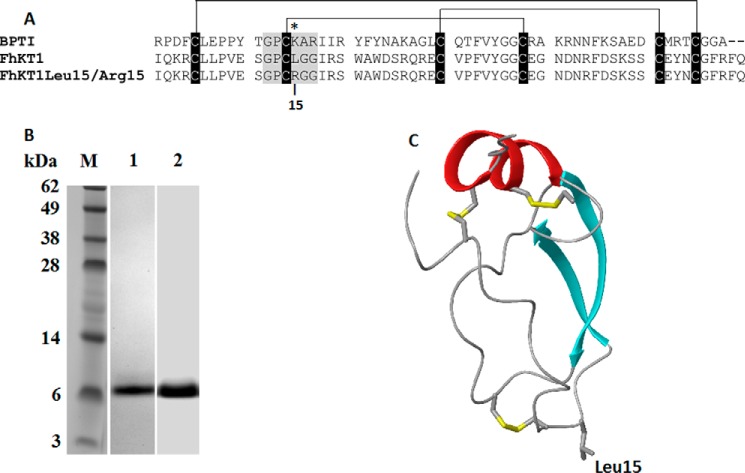
**Structural representation of FhKT1 and FhKT1Leu^15^/Arg^15^ and their recombinant expression.** *A,* sequence alignment of BPTI, FhKT1, and FhKT1Leu^15^/Arg^15^. The *asterisk* denotes the P1 site at position 15. *Lines* indicate the conserved disulfide bonds that occur between Cys^1^ and Cys^6^, Cys^2^ and Cys^4^, and Cys^3^ and Cys^5^, with cysteine residues highlighted in *black bars. B,* recombinant forms of FhKT1 and FhKT1Leu^15^/Arg^15^ were expressed as secretory proteins in the methylotrophic yeast *P. pastoris* with a yield of ∼5–10 mg of soluble protein from each 1 liter of culture. rFhKT1 (*lane 1*) and rFhKT1Leu^15^/Arg^15^ (*lane 2*) were isolated by NTA-affinity chromatography and analyzed by NuPAGE Novex 4–12% BisTris protein gel, stained with Biosafe Coomassie (Bio-Rad). *C,* homology model of FhKT1 built based on BPTI (PDB code 3OTJ) displaying the three disulfide bonds (*yellow*), the α-helix (*red*), and anti-parallel β-sheets (*blue*) characteristic of KT protease inhibitors. The P1 Leu^15^ located at the peak of the reactive loop is also shown in *gray*.

**FIGURE 3.**
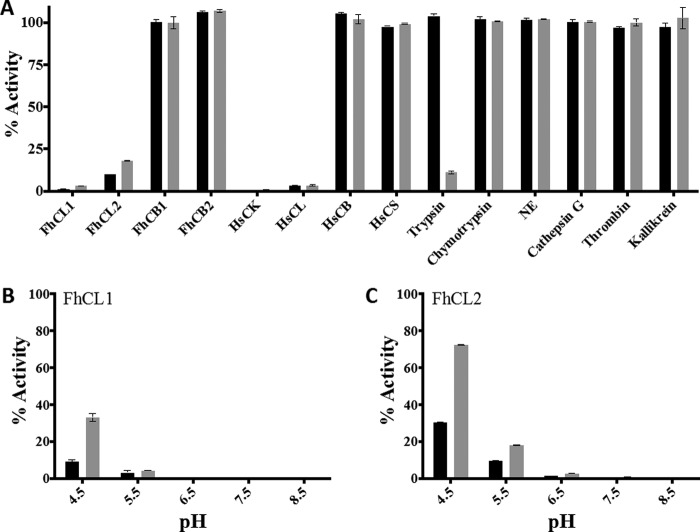
**Inhibition profile of FhKT1 and FhKT1Leu^15^/Arg^15^ against cysteine and serine proteases.** *A,* inhibitory activity of rFhKT1 (*black bars*) and rFhKT1Leu^15^/Arg^15^ (*gray bars*) at 300 nm was screened using a range of cysteine proteases, including *F. hepatica* cathepsin L1 (*FhCL1*), *F. hepatica* cathepsin L2 (*FhCL2*), *F. hepatica* cathepsin B1 (*FhCB1*), *F. hepatica* cathepsin B2 (*FhCB2*), human cathepsin K (*HsCK*), human cathepsin L (*HsCL*), human cathepsin B (*HsCB*), human cathepsin S (*HsCS*), and serine proteases, including trypsin, chymotrypsin, neutrophil elastase (*NE*), cathepsin G, thrombin, and kallikrein. Inhibition is presented relative to the activity of each enzyme in the absence of inhibitors. *B,* activity of FhCL1 in the presence of 300 nm rFhKT1 (*black bars*) and rFhKT1Leu^15^/Arg^15^ (*gray bars*) at different pH values (see “Experimental Procedures” for details of buffer systems) relative to FhCL1 activity in the absence of inhibitors. *C,* activity of FhCL2 in the presence of 300 nm rFhKT1 (*black bars*) and rFhKT1Leu^15^/Arg^15^ (*gray bars*) at different pH values, relative to FhCL2 activity in the absence of inhibitors. *Error bars* indicate standard deviation of three separate experiments.

**FIGURE 4.**
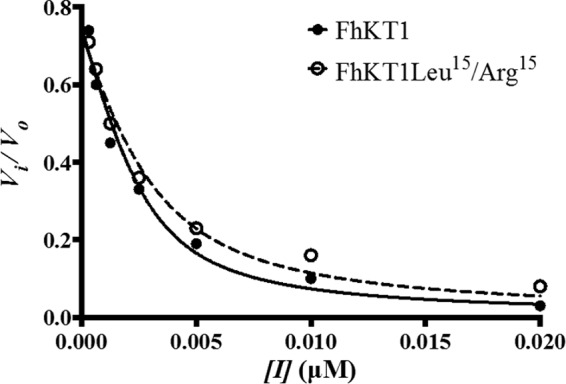
**Plot of *F. hepatica* cathepsin L1 proteolytic activity with the substrate Z-Leu-Arg-NHMec, as a function of inhibitor concentration for rFhKT1 (*solid circles*) and rFhKT1Leu^15^/Arg^15^ (*clear circles*).** The *curves* represent the best fit to the Morrison equation (*solid line* = rFhKT1; *dashed line* = rFhKT1Leu^15^/Arg^15^). *Error bars* indicate standard deviation of three separate experiments.

Surrogate expression of the *fhkt1* gene in the yeast *Pichia pastoris* allowed us to produce and purify a recombinant form of the inhibitor (*rFhKT1,* Fig. 2). Surprisingly, in our initial screens we found that the purified rFhKT1 did not exhibit any inhibition against a variety of standard serine proteases, including trypsin, chymotrypsin, elastase, thrombin, kallikrein, and human cathepsin G (Fig. 3*A*). This was not due to the recombinant protein being functionally inactive as we subsequently discovered that rFhKT1 was a potent inhibitor of two *F. hepatica* cathepsin L-like cysteine proteases, FhCL1 and FhCL2. In further screens, rFhKT1 was shown to inhibit human cathepsin L and cathepsin K (Fig. 3*A*). In these initial inhibition screens, rFhKT1 was tested at 300 nm, and the activity of FhCL1, FhCL2, and human cathepsins K and L was reduced by 98.86% (±0.205), 90.29% (±0.025), 99.54% (±0.08), and 96.79% (±0.09), respectively (Fig. 3*A*). Of further relevance was our observations that at a 300 nm final concentration rFhKT1 did not show any inhibition of *F. hepatica* cathepsin B1 (FhCB1) and B2 (FhCB2) or of human cathepsin B and cathepsin S (Table 1). In parallel assays, we showed that the activity of trypsin, chymotrypsin, neutrophil elastase, and cathepsin G was completely inhibited by 300 nm BPTI and that thrombin and kallikrein were completely inhibited by 0.002 units of hirudin (data not shown). Given that the concentration of FhKT1 inhibitor required for the inhibition of the cathepsin L and K cysteine proteases was found to be lower than the concentration of enzyme required to produce a reliable activity curve in fluorogenic assays, these were considered to be tight-binding inhibitors, and thus enzymatic data were fitted to the Morrison equation (Fig. 4). Resulting *K*_*i*_^app^ values were fitted to Equation 2 mentioned above to determine *K_i_* values. Kinetic inhibition constants determined at pH 5.5 for rFhKT1 against the two parasite proteases differed substantially; the *K_i_* for FhCL1 was 0.4 (±0.1) nm and for FhCL2 was 10 (±0.3) nm. The inhibition constant was 1.6 (±0.1) nm for human cathepsin L and 5 (±0.3) nm for human cathepsin K (Table 1).

**TABLE 1:** **rFhKT1 and rFhKT1Leu^15^/Arg^15^ inhibition specificity against a panel of biologically relevant serine and cysteine proteases and relative inhibition constants**

Enzyme (concentration)	rFhKT1		rFhKT1Leu^15^/Arg^15^	
	Inhibition	*K_i_*	Inhibition	*K_i_*
**Cysteine proteases**				
*F. hepatica* cathepsin L1 (2.7 nm)	+	0.4 nm (± 0.1 nm)	+	0.7 nm (± 0.04 nm)
*F. hepatica* cathepsin L2 (5 nm)	+	10 nm (± 0.3 nm)	+	27 nm (± 1.5 nm)
*F. hepatica* cathepsin B1 (180 nm)	−	Not inhibited	−	Not inhibited
*F. hepatica* cathepsin B2 (180 nm)	−	Not inhibited	−	Not inhibited
Human cathepsin L (0.2 nm)	+	1.6 nm (± 0.1 nm)	+	3 nm (± 0.1 nm)
Human cathepsin K (2 nm)	+	5 nm (± 0.3 nm)	+	10 nm (± 0.2 nm)
Human cathepsin S (2 nm)	−	Not inhibited	−	Not inhibited
Human cathepsin B (3.6 nm)	−	Not inhibited	−	Not inhibited

**Serine proteases**				
Trypsin (42 nm)	−	Not inhibited	+	1.5 nm (± 0.7 nm)
Chymotrypsin (40 nm)	−	Not inhibited	−	Not inhibited
Neutrophil elastase (50 nm)	−	Not inhibited	−	Not inhibited
Cathepsin G (100 nm)	−	Not inhibited	−	Not inhibited
Thrombin (43 pm)	−	Not inhibited	−	Not inhibited
Kallikrein (150 nm)	−	Not inhibited	−	Not inhibited

##### Impact of Substituting the FhKT1 P1 Leu Residue to Arg on Protease Inhibition

Given the importance of the P1 residue at position 15 within the exposed loop in KT protease inhibitors, we examined the impact of substituting this residue in FhKT1 from a leucine to the more commonly found arginine. This variant form, termed rFhKT1Leu^15^/Arg^15^, retained its ability to inhibit *F. hepatica* FhCL1 and FhCL2 and human cathepsin L and K, but as with the wild-type rFhKT1, it did not inhibit FhCB1 and FhCB2 and human cathepsin B and S (Table 1). However, several key observations were made, including the following: (*a*) rFhKT1Leu^15^/Arg^15^ was less inhibitory than rFhKT1 with *K_i_* 2–3-fold higher against the parasite and mammalian cysteine proteases (Table 1); (*b*) although the inhibition of FhCL1 and FhCL2 by both rFhKT1 and rFhKT1Leu^15^/Arg^15^ was effective over a broad pH range of 4.5–8.0 and optimal at neutral pH, the variant inhibitor was less effective in the lower pH range (Fig. 3, *B* and *C*); (*c*) in contrast to rFhKT1, the variant rFhKT1Leu^15^/Arg^15^ possessed potent inhibitory activity against the serine protease trypsin, with trypsin activity reduced by 88.92% (±0.89) in assays performed at 300 nm inhibitor (Fig. 3*A*). rFhKT1Leu^15^/Arg^15^ exhibited a *K_i_* of 1.5 nm (±0.7 nm) against trypsin (Table 1), which demonstrates that the substitution of Leu to Arg is sufficient to endow this inhibitor with the ability to bind within the active site of this serine protease. However, it is noteworthy that this inhibitory constant is still 10 times higher than that recorded for BPTI (0.15 nm), and even at 300 nm, rFhKT1Leu^15^/Arg^15^ did not show any inhibition toward the other serine proteases tested, including chymotrypsin, elastase, thrombin, kallikrein, and human cathepsin G (Fig. 3*A*).

##### rFhKT1 Prevents the Auto-catalytic Activation of Parasite Cysteine Proteases

**FIGURE 5.**
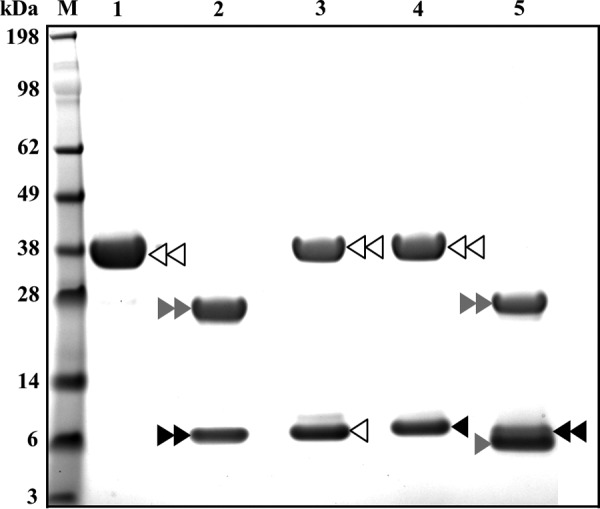
**Inhibition of the auto-catalytic activation of FhCL1 by rFhKT1 and rFhKT1Leu^15^/Arg^15^.** *M,* molecular mass marker; *lane 1,* FhCL1 zymogen; *lane 2,* mature FhCL1 following auto-catalytic activation at pH 4.5 for 1 h; *lane 3,* FhCL1 and rFhKT1 following incubation at pH 4.5 for 1 h; *lane 4,* FhCL1 and rFhKT1Leu^15^/Arg^15^ following incubation at pH 4.5 for 1 h; *lane 5,* FhCL1 and bovine pancreatic trypsin inhibitor following incubation at pH 4.5 for 1 h. *Double white arrowhead* indicates FhCL1 zymogen; *double gray arrowhead* indicates mature FhCL1, and *double black arrowhead* indicates FhCL1 pro-peptide removed following auto-catalytic activation. *Single white arrowhead* indicates position of FhKT1; *single black arrowhead* indicates FhKT1Leu^15^/Arg^15^, and *single gray arrowhead* shows position of BPTI. Proteins were separated on a NuPAGE Novex 4–12% BisTris protein gel and stained with Biosafe Coomassie (Bio-Rad). *M,* molecular mass markers.

The parasite cysteine proteases FhCL1 and FhCL2 are produced as inactive 37-kDa zymogens (Fig. 5, *lane 1*) that can be auto-catalytically activated *in vitro* at pH 4.5 (34). To determine whether rFhKT1 and rFhKT1Leu^15^/Arg^15^ could regulate the activation of the parasite proteases, these inhibitors were mixed with zymogens of FhCL1 and FhCL2 at pH 4.5, and the activation was monitored by LDS-PAGE. After 45 min of incubation at 37 °C in citrate phosphate buffer, pH 4.5, FhCL1 auto-activates to give rise to an ∼25-kDa mature active form and a released 6-kDa propeptide (Fig. 5, *lane 2*). Incubation of FhCL1 with rFhKT1 or rFhKT1Leu^15^/Arg^15^ prevented the appearance of both of these bands indicating that auto-catalytic cleavage was prevented (Fig. 5, *lanes 3* and *4*). Similar results were observed for FhCL2 (data not shown). For comparison, we showed that co-incubation of FhCL1 with the KT inhibitor BPTI did not prevent auto-catalytic activation of the zymogen (Fig. 5, *lane 5*).

**FIGURE 6.**
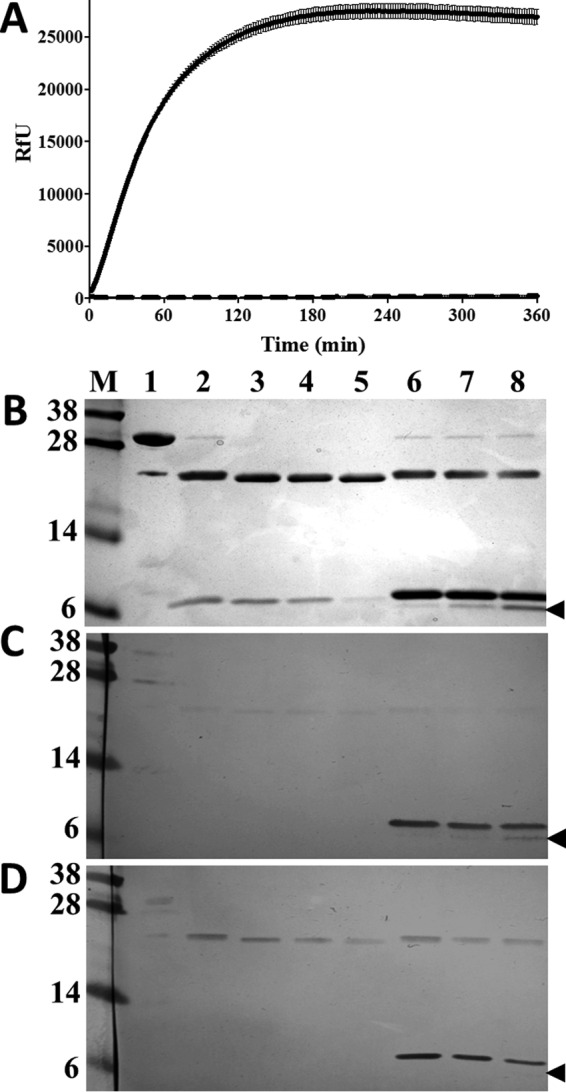
**rFhKT1 inhibits mature FhCL1 and forms a stable complex.** *A,* progress curve of fully activated FhCL1 inhibited by rFhKT1 over 360 min. Activity is represented by relative fluorescent units. Activity of uninhibited FhCL1 is represented by the *solid line*, and rFhKT1-inhibited FhCL1 is represented by the *dashed line. Error bars* indicate standard deviation of three separate experiments. *B,* FhCL1 zymogen (*lane 1*) was auto-catalytically activated at pH 4.5 for 1 h to the mature active enzyme (*lane 2*). The mature enzyme was then incubated in the absence (*lanes 3–5*) or presence (*lanes 6–8*) of rFhKT1 for 1 h (*lanes 3* and *6*), 2 h (*lanes 4* and *7*), and 6 h (*lanes 5* and *8*), and then the reaction mixes were analyzed by LDS-PAGE. The protein gel was stained with Biosafe Coomassie (Bio-Rad). Replicate gels were used for Western blot analysis by probing with polyclonal anti-rFhKT1 (*C*) and polyclonal antibody to His_6_-tagged proteins polyclonal antibodies (*D*). The *arrowhead* indicates the position of a cleaved product of ∼5-kDa band that reacts with polyclonal anti-rFhKT1 but not anti-His_6_-tagged antibodies indicating that it represents a cleavage product of rFhKT1. *M,* molecular mass markers.

To examine the stability of inactivation of FhCL1 by rFhKT1 over time, FhCL1 was first auto-catalytically activated at pH 4.5 and then incubated with rFhKT1 for 6 h at 37 °C in citrate phosphate buffer, pH 5.5, in the presence of substrate; the reaction was monitored by the release of the fluorophore (7-amino-4-methylcoumarin), and samples of the reactions were analyzed by LDS-PAGE (Fig. 6). rFhKT1 completely inactivated the FhCL1, and this inactivation was stable for the entire 6 h (Fig. 6*A*). Furthermore, the rFhKT1 remained intact over this time and was observed as a band at ∼6 kDa. However, a minor band at ∼5 kDa was observed at the 2- and 6-h time points (Fig. 6*B*). This band was not immunoreactive with anti-His tag monoclonal antibodies (Fig. 6*D*), but it was reactive with anti-rFhKT1 polyclonal antibodies (Fig. 6*C*), showing that this protein results from proteolytic clipping at the C terminus of rFhKT1 by FhCL1. A similar cleavage pattern was observed with rFhKT1Leu^15^/Arg^15^ and also when FhCL2 was used in the reactions (data not shown).

Inhibition assays that were extended for up to 7 days showed that FhCL1 activity remained inhibited by rFhKT1 over this prolonged period. However, the activity of mature FhCL1 (in the absence of inhibitor) depleted by >99% over the course of the experiment, with activity reduced from 21,271.27 (± 961.52) RfU/min at 0 days to 65.73 (± 14.49) RfU/min at day 7 (data not shown).

##### Inhibitory Activity of rFhKT1 Is Dependent on Internal Disulfide Bridges

**FIGURE 7.**
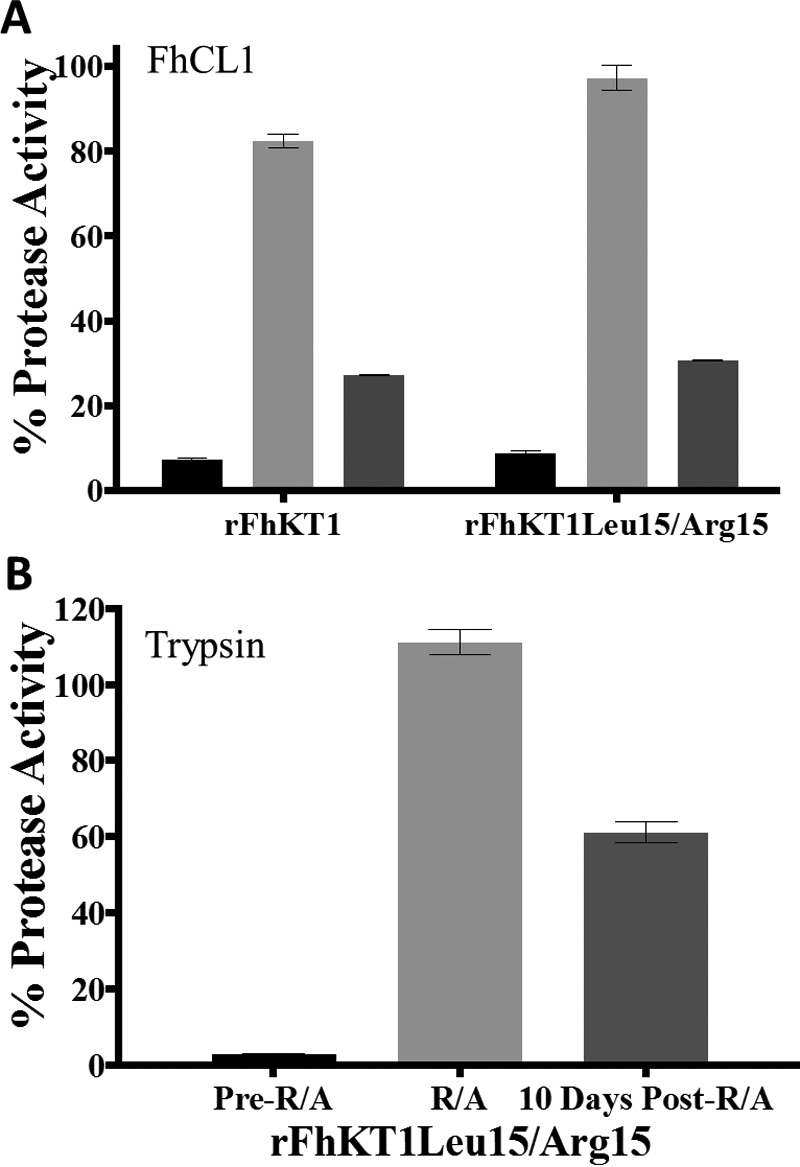
**Inhibitory activity of rFhKT1 and rFhKT1Leu^15^/Arg^15^ is dependent on disulfide bridges.** *A,* inhibition of FhCL1 activity by rFhKT1 and rFhKT1Leu^15^/Arg^15^ before (*black bars*) and after reduction (*R*) and alkylation (*A*) (*light gray bars*) and with reduced and alkylated inhibitor following incubation for 10 days at 4 °C (*dark gray bars*). *B,* inhibition of trypsin activity by rFhKT1Leu^15^/Arg^15^ before (pre-reduction and alkylation (*R/A*), *black bars*) and after reduction and alkylation (R/A, *light gray bars*), and with reduced and alkylated inhibitor following incubation for 10 days at 4 °C (post-R/A, *dark gray bars*). *Error bars* indicate standard deviation.

Reduction and alkylation of both rFhKT1 and rFhKT1Leu^15^/Arg^15^ were performed to break internal disulfide bridges and to disrupt the tertiary structure of the protein. This procedure depleted the inhibitory activity of both inhibitors against FhCL1 (Fig. 7*A*). Similarly, this treatment ablated the inhibitory activity of rFhKT1Leu^15^/Arg^15^ against trypsin (Fig. 7*B*). Interestingly, we discovered that both rFhKT1 and rFhKT1Leu^15^/Arg^15^ could partially re-nature to their functional form during a 10-day incubation period at 4 °C; over this time, the inhibitors regained between 60 and 80% of their inhibitory activity (Fig. 7*A*). Likewise, rFhKT1Leu^15^/Arg^15^ regained inhibitory activity against trypsin (Fig. 7*B*).

##### rFhKT1 Interacts with Several Native F. hepatica Cysteine Proteases

**FIGURE 8.**
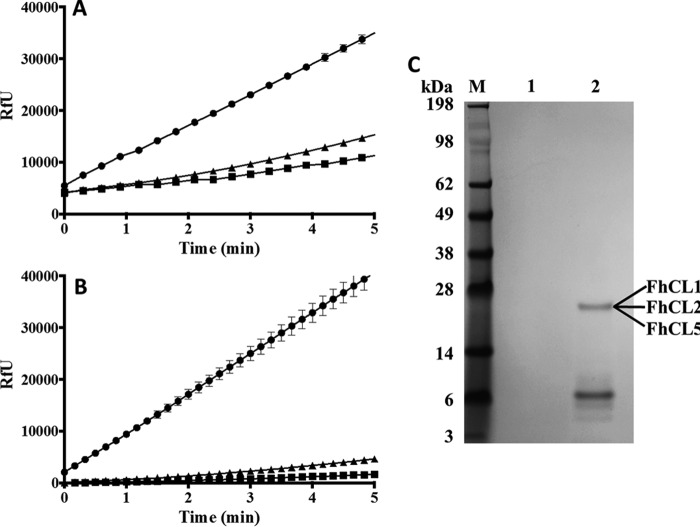
**rFhKT1 binds and inhibits native *F. hepatica*-secreted cysteine proteases.** *A,* cysteine protease activity (presented as RfU) in adult *F. hepatica* RPMI 1640 culture media measured with the fluorogenic peptide substrate, Z-Phe-Arg-NHMec, in the absence (*circles*) or presence of rFhKT1 (*squares*) or rFhKT1Leu^15^/Arg^15^ (*triangles*). *B,* cysteine protease activity in the adult *F. hepatica* RPMI 1640 culture media following pulldown using Ni-NTA beads alone (*circles*), rFhKT1 and Ni-NTA beads (*squares*), or rFhKT1Leu^15^/Arg^15^ and Ni-NTA beads (*triangles*). *C,* LDS-PAGE analysis of pulldown from adult *F. hepatica* RPMI 1640 culture media with Ni-NTA beads alone (*lane 1*) and with rFhKT1 and Ni-NTA beads (*lane 2*), stained with Biosafe Coomassie (Bio-Rad). LC-MS/MS analysis of the 26-kDa band observed in *lane 2* revealed that it consisted of FhCL1, FhCL2, and FhCL5 in their fully active mature forms. *M,* molecular mass marker.

Adult *F. hepatica* parasites secrete several cysteine proteases, both *in vivo* and *in vitro*. To demonstrate that FhKT1 can bind and inhibit native parasite proteases, the inhibitors were mixed with culture medium in which adult *F. hepatica* parasites had been maintained (*F. hepatica* excretory-secretory proteins), and activity was monitored using Z-Phe-Arg-NHMec as substrate; both inhibitors blocked >80% of total secreted cathepsin L-like activity (Fig. 8*A*).

To identify the native cysteine proteases to which rFhKT1 bound, this inhibitor was added to media in which adult parasites were cultured for 5 h. Following the parasite culture, the media were removed, and rFhKT1 was pulled down with Ni-NTA beads; fluorogenic assays demonstrated that removal of the inhibitors from each medium, respectively, also resulted in the depletion of >95% of the cysteine protease activity secreted by the adult parasites (Fig. 8*B*). LDS-PAGE analysis of the proteins bound to the Ni-NTA beads revealed a 6-kDa band representing the rFhKT1 and another band of ∼25 kDa, which is in keeping with the size of mature cathepsin L cysteine proteases (Fig. 8*C*). The 6- and ∼25-kDa proteins were not detected when Ni-NTA beads were added to the culture media containing no recombinant inhibitor. LC-MS/MS analysis verified that the ∼25-kDa band pulled down by rFhKT1 consisted of a mix of *F. hepatica* cysteine proteases in their mature forms, including FhCL1, FhCL2, and another cathepsin L protease known as FhCL5 (supplemental file 2) (6, 35).

##### FhKT1 Binds to the Cysteine Protease Active Site in a Unique Manner

**FIGURE 9.**
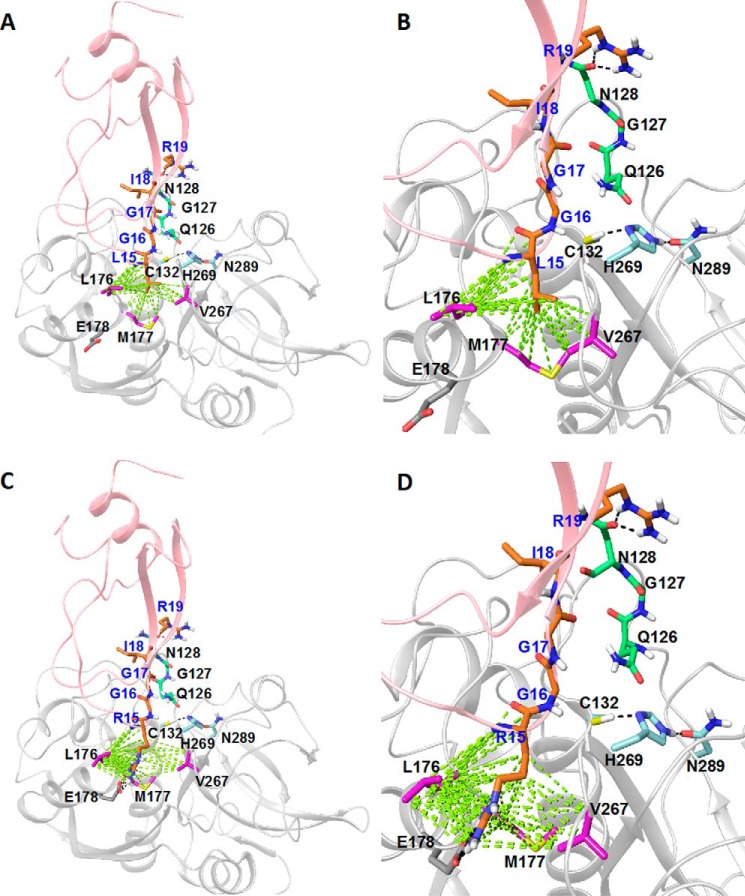
**Molecular model of FhKT1-*F. hepatica* cathepsin L1 interactions.** The homology model of FhKT1 (*A* and *B*) and FhKT1Leu^15^/Arg^15^ (*C* and *D*) built based on BPTI (PDB code 3OTJ) and the crystal structure of *F. hepatica* cathepsin L (PDB code 2O6X) are shown in *pink* and *gray schematics*, respectively. The interacting residues of FhKT1 and FhCL1, the residues of the FhCL1 S1 pocket (Cys^132^, His^269^, and Asn^289^), and the residues of the S2′ pocket (Gln^126^, Gly^127^, and Asn^128^) and Glu^178^ are visualized in *stick-like* representation. The residues of the S2 pocket, the residues of S2′ pocket, and other interacting residues of FhCL1 are shown in *purple, green,* and *gray*, respectively. Hydrogen bonds and hydrophobic interactions are in *black* and *green,* respectively. Residue labels are in *blue* for FhKT1 and *black* for FhCL1. *A* and *B,* Leu^15^ of FhKT1 is predicted to sit in the hydrophobic S2 pocket of FhCL1, forming contacts with Leu^176^, Met^177^, and Val^267^. In addition, it is indicated that FhKT1 forms H-bonds with Asn^128^ of the S2′ pocket. It is also predicted that Gly^16^ and Gly^17^ block substrate access to the S1 active site pocket by being positioned across it, without actually penetrating the pocket. *C* and *D,* Arg^15^ of FhKT1Leu^15^/Arg^15^ is predicted to sit in the hydrophobic S2 pocket of FhCL1, forming contacts with Leu^176^, Met^177^, and Val^267^. The guanidine group of Arg^15^ is predicted to interact with Glu^178^ via H-bonds. In addition, as for FhKT1 it is also indicated that Gly^16^ and Gly^17^ sit across the S1 active site pocket without penetrating the pocket itself, thus blocking substrate access. FhKT1Leu^15^/Arg^15^ is also predicted to form H-bonds with Asn^128^ of the S2′ pocket. *Panels* at *right* are enlarged versions of the *left*, with focus toward the FhCL1 active site.

To understand the molecular basis of FhKT1 binding to cathepsins, a homology model of FhKT1 was built using the crystal structure of BPTI as a template. Although protein sequence identity between BPTI and FhKT1 is only 23%, both possess the six conserved cysteine residues that form three disulfide bonds (11), suggesting similar folding (root mean square deviation = 1.3 Å). The homology model of FhKT1 was docked to the crystal structure of FhCL1 previously reported by us (36). The rigid body protein-protein docking protocol was employed, and the selected docking solution was optimized using molecular dynamic simulations. A docking solution in which the P1 Leu^15^-containing side of FhKT1 faces the active site of FhCL1, similar to the BPTI binding orientation in trypsin in the crystal structure, was used for optimization. The resultant protein-protein complex indicates that FhKT1 binds sufficiently tight within the active site groove of FhCL1 to block access of small substrates (*e.g.* Z-Phe-Arg-NHMec) to the reactive cysteine (Cys^132^) in the S1 active site pocket (Fig. 9, *A* and *B*).

**FIGURE 10.**
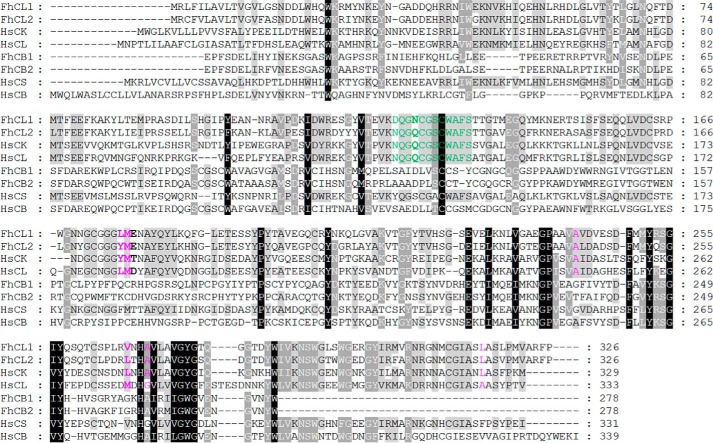
**Sequence alignment of *F. hepatica* and *Homo sapiens* cysteine proteases.** *F. hepatica* cysteine protease sequences include FhCL1 (cathepsin L1, AAB41670), FhCL2 (cathepsin L2, AAC47721), FhCB1 (cathepsin B1, ABF85678), and FhCB2 (cathepsin B2, ABF85679). *H. sapiens* cysteine protease sequences include HsCK (cathepsin K, NP_000387), HsCL (cathepsin L, NP_001903), HsCS (cathepsin S, NP_004070), and HsCB (cathepsin B, NP_001899). Residues surrounding the S1 active site region are shown in *green*, and residues forming the S2 active site are shown in *pink*. Residues that form interactions with FhKT1 and FhKT1Leu^15^/Arg^15^ are shown in *bold*.

In this model of interactions, the residue Leu^15^ of the exposed loop of FhKT1 forms strong hydrophobic interactions with residues Leu^176^, Met^177^, and Val^267^ of the S2 pocket of FhCL1 (Fig. 9, *A* and *B*). This places Gly^16^ and Gly^17^ across the S1 pocket, but neither penetrates this subsite. The Ile^18^ is not predicted to make any interactions with the FhCL1, but Arg^19^ forms an H-bond with the Asn^128^ of the S2′ pocket. Relevantly, the sequence alignment of the cysteine proteases (Tables 2 and 3 and Fig. 10) used in this study shows that residues of the S2 and S1′-S2′ pockets are highly similar in physicochemical properties in FhCL1, FhCL2, human cathepsins L and K, which are all inhibited by FhKT1. By contrast, these residues are diverse in *F. hepatica* cathepsins FhCB1 and FhCB2, and in human cathepsins B and S, which are not inhibited by FhKT1 (Tables 2 and 3 and Fig. 10). This observation suggests that the S2 and S1′-S2′ pockets play a crucial role in selective binding of FhKT1 to FhCL1, FhCL2, and human cathepsins L and K.

**TABLE 2:** **Comparison of residues forming the S2 active site of cysteine proteases from *F. hepatica* and *H. sapiens***

	S2 residues*^a^*					
	176	177	242	267	270	316
*F. hepatica* CL1	Leu	Met	Ala	Val	Ala	Leu
*F. hepatica* CL2	Tyr	Met	Ala	Leu	Ala	Leu
Human cathepsin K	Tyr	Met	Ala	Leu	Ala	Leu
Human cathepsin L	Leu	Met	Ala	Met	Gly	Ala
*F. hepatica* CB1	Gln	Cys	Gly	Gly	Ala	N/A
*F. hepatica* CB2	Lys	Cys	Thr	Gly	Ala	N/A
Human cathepsin S	Phe	Met	Gly	Val	Gly	Phe
Human cathepsin B	Pro	Cys	Ala	Gly	Ala	Val

*^a^* Residue numbering is based on FhCL1 (shown in *pink* in Fig. 10).

**TABLE 3:** **Comparison of residues surrounding the S1 active site region of cysteine proteases from *F. hepatica* and *H. sapiens* (based on sequence alignment)**

	Residue no.*^a^*											
	125	126	127	128	129	130	131	132*^b^*	133	134	135	136
*F. hepatica* CL1	Asp	Gln	Gly	Asn	Cys	Gly	Ser	**Cys**	Trp	Ala	Phe	Ser
*F. hepatica* CL2	Asp	Gln	Gly	Gln	Cys	Gly	Ser	**Cys**	Trp	Ala	Phe	Ser
Human cathepsin K	Asn	Gln	Gly	Gln	Cys	Gly	Ser	**Cys**	Trp	Ala	Phe	Ser
Human cathepsin L	Asn	Gln	Gly	Gln	Cys	Gly	Ser	**Cys**	Trp	Ala	Phe	Ser
*F. hepatica* CB1	Ser	Ala	Ile	Asp	Leu	Val	Ser	**Cys**	Cys	Ser	Tyr	Cys
*F. hepatica* CB2	Ala	Ala	Ala	Asp	Pro	Leu	Ser	**Cys**	Cys	Thr	Tyr	Cys
Human cathepsin S	Tyr	Gln	Gly	Ser	Cys	Gly	Ala	**Cys**	Trp	Ala	Phe	Ser
Human cathepsin B	Ser	Ala	Glu	Asp	Leu	Leu	Thr	**Cys**	Cys	Gly	Ser	Met

*^a^* Residue numbering is based on FhCL1.

*^b^* Active site cysteine residues are shown in boldface.

The binding orientation we obtained for the P1 Leu^15^ helps explain the interactions of Arg at this position in the variant rFhKT1Leu^15^/Arg^15^. The hydrophobic tail of Arg^15^ can form hydrophobic interactions with the S2 pocket, whereas the guanidine group could form H-bonds with Glu^178^ of FhCL1 (Fig. 9, *C* and *D*). Intriguingly, the other cathepsins that we have shown to bind FhKT1Leu^15^/Arg^15^, *i.e.* FhCL2 and human cathepsins L and K, also have a residue capable of forming H-bonds at this position (Table 2 and Fig. 10).

Unlike BPTI, FhKT1 does not bind to trypsin and other serine proteases tested in this study. In the available crystal structures of the BPTI-trypsin complex, the P1 Lys^15^ of BPTI forms H-bonds with Asp^194^ and Ser^195^ of the trypsin S1 (37). However, the hydrophobic P1 Leu^15^ of FhKT1 is unlikely to bind to the hydrophilic S1 pocket of serine proteases preventing the formation of the protein-protein complex. Binding of rFhKT1Leu^15^/Arg^15^ to trypsin, however, suggests that the P1 Arg^15^ can form these crucial H-bonds in the S1 pocket.

Kunitz inhibitors with P1 Leu often exhibit an increased specificity for chymotrypsin over trypsin (11). In this study, however, we found that FhKT with a P1 Leu^15^ does not inhibit chymotrypsin. Our modeling of these two molecules suggests that this could be due to different structuring of surface polypeptide loops of FhKT1 and chymotrypsin that are unable to establish a complementary interface to form a complex and allow the P1 Leu^15^ to enter the active site.

## Discussion

We have shown that the infective stage of the helminth parasite *F. hepatica* expresses a temporally regulated and secreted Kunitz-type (KT) protease inhibitor (FhKT1) with novel inhibition properties. Unlike any previously described KT protease inhibitors, FhKT1 exhibits exclusive and stable inhibition of cysteine proteases, specifically cathepsin L- and K-like cysteine proteases. Recombinant FhKT1 inhibits these cysteine proteases across a wide pH range, from pH 4.5 to 8.5, with optimal inhibition at neutral pH. Our studies suggest that the six conserved cysteine residues that maintain the structural integrity of the central domain of KT-type inhibitors perform a similar function in FhKT1 as reduction and alkylation of the disulfide cross-links abrogate inhibitory activity.

Previously, we resolved the three-dimensional structure of FhCL1 and showed that the active site is typical of the cathepsin L cysteine proteases, consisting of a deep open groove created between the right and left domains of the enzyme (36). Here, we built a homology model of FhKT1 on the basis of the known BPTI structure and docked it into the FhCL1 active site to provide insights into the interactions responsible for the binding of FhKT1 to cathepsin L-like proteases. The P1 Leu^15^ in FhKT1, which projects from the peak of the inhibitor reactive loop, is predicted to form crucial hydrophobic interactions with residues in the FhCL1 S2 active site pocket (Leu^176^, Met^177^, and Val^267^), whereas residue Arg^19^ of FhKT1 is predicted to form H-bond interactions with Asn^128^ within the S2′ pocket, respectively. Because FhKT1 spans these S and S′ subsites, it places the Gly^16^ and Gly^17^ residues in a position whereby they block access of even small molecules, such as Z-Phe-Arg-NHMec, to the reactive Cys^132^ residue in the S1 pocket that is essential for forming a thiol intermediate with the substrate before the scissile bond is cleaved.

It is intriguing that FhKT1 also exhibits specificity within the cathepsin-like cysteine proteases themselves by not inhibiting parasite or human cathepsin B and human cathepsin S. This specificity of inhibition was explained by our computational modeling and sequence comparison that revealed a distinct difference in the composition of the S2 and S1′-S2′ active site pockets within this group of proteases; these changes reduce their hydrophobicity and alter charge arrangements such that they cannot interact with the P1 Leu^15^ (see Tables 2 and 3 and Fig. 10). It is noteworthy that the occluding loop that is common in cathepsin B proteases and is responsible for conferring these enzymes with carboxypeptidase activity (38) does not prevent access of FhKT1 to the active site.

Although the P1 Leu^15^ of FhKT1 was predicted to be important for interactions with the hydrophobic S2 pocket of the cathepsin L-like proteases, this residue could be replaced by Arg (FhKT1Leu^15^/Arg^15^). The P1 Arg^15^ did exert some adverse effects, especially at low pH, on the inhibitor's activity, but it did not alter its specificity against cysteine proteases. Molecular docking with FhCL1 indicated that the hydrophobic tail of Arg^15^ could still form hydrophobic interactions with the S2 pocket, similar to those predicted for Leu^15^. In addition, the guanidine group of Arg^15^ is predicted to form H-bonds with Glu^178^. These interactions could not take place in the cathepsin B and S proteases, and therefore, the variant showed no activity against these enzymes. Other amino acid replacements at position 15, however, could likely have a more dramatic effect on the FhKT1's inhibitory properties.

There are no previous reports of a KT-type protease inhibitor that exclusively inhibits cysteine proteases like FhKT1, although there are several that describe members with low cross-activity against other proteases. For example, a KT inhibitor from the hard tick *Boophilus microplus* (BmTIsint) blocks serine proteases (trypsin and plasma kallikrein, *K_i_* = 3.3 and 16.5 nm, respectively), as well as the cysteine protease human cathepsin L (*K_i_* = 108 nm) (39), and the KT inhibitor (ShPI-1A) from the sea anemone *Stichodactyla helianthus* exhibits broad inhibitory specificity toward serine (trypsin *K_i_* = 0.1 nm, chymotrypsin *K_i_* = 2.3 nm, human plasmin *K_i_* = 2.7 nm, and porcine kallikrein *K_i_* = 85 nm), cysteine (*K_i_* for papain not determined), and aspartic proteases (porcine pepsin *K_i_* = 33 nm) (40, 41). Like rFhKT1Leu^15^/Arg^15^, these KT inhibitors possess at P1 Arg^15^ that accounts for their serine protease activity; however, apart from BmTIsint having a Glu^39^ residue at a similar position to FhKT1, it is not possible to fully explain their cysteine protease inhibitory activity from primary sequence comparisons alone (39). Interestingly, a KT inhibitor reported from the blood feeding horn fly *Hematobia irritans irritans* has a P1 Arg^15^ and residues Glu^10^ and Arg^44^ similar to FhKT1 as well as an uncommon residue at position 39 (His^39^) (42), which implies that it could inhibit both serine and cysteine proteases. Clarification of the residues involved in restricted and broad protease inhibition will emerge as this and other KT inhibitors are more fully characterized and will inform the development of methods for functional predictions. For now, however, we suggest that KT inhibitors should be classified into subgroups based on their inhibition profile, including KT serine-specific protease inhibitors, KT cysteine-specific protease inhibitors, KT serine/cysteine protease inhibitors, and even KT serine/cysteine/aspartic protease inhibitors. Our study also points toward the opportunity of designing specific and broad inhibitors against proteases of various mechanistic classes by making defined alterations to the basic 68-amino acid scaffold of the KT inhibitor.

To facilitate penetration of the host intestine and migration through the liver, the infective juvenile parasite secretes an array of temporally regulated cathepsin L proteases (36). We have shown that the expression of FhKT1 is also highly up-regulated in the first 24 h after excystment. Protein expression was shown by immunolocalization studies to be associated most particularly with cells bodies within the parenchymal tissue. The parenchymal tissues fill the spaces between the internal organs of the parasite and are suggested to function as a flexible cytoskeleton and an important transport system (43, 44). The cell bodies, which are separated from each other within the parenchymal tissue, are highly metabolically active and contain numerous mitochondria and Golgi complexes (44, 45). They act as a store for carbohydrate reserves, such a glycogen, and are important in parasite metabolism and growth as they become depleted as the parasite develops in the first 24 h (46). The cell bodies have contacts with the gastrodermal cells lining the parasite gut where we detected more diffuse labeling of the FhKT1. The up-regulation of the FhKT1 in the cell bodies and gut correlates with the parasite's penetration of the duodenum wall after excystment, which is facilitated by the secretion of gut cysteine proteases suggesting that the inhibitor plays a role in controlling proteolytic activity at this crucial phase of infection.

We also showed by pulldown experiments that FhKT1 binds and inactivates native FhCL1, FhCL2, and FhCL5 secreted by the adult parasite and thus has broad specificity for the parasite's cathepsin Ls. Importantly, FhKT1 prevents the auto-catalytic activation of the inactive cathepsin zymogen to the mature active enzyme. In a similar manner to the function of BPTI, which regulates trypsin activation and activity to prevent excessive tissue damage in the pancreas (47), FhKT1 may control the activation of the parasite-secreted cathepsin Ls, preventing aberrant activation within the parasite itself (9, 48). Limiting cysteine protease activity would be important to the parasite's survival in the host, because although the enzymes are necessary for degrading host blood and tissues, they also make significant contributions to the clinical manifestations of the disease, including tissue damage, hemorrhaging, and induction of inflammatory immune responses. By complexing and inactivating excess secreted proteases, FhKT1 could also restrict this pathology and prevent transport of damaging proteases via the circulation that could cause tissue inflammation at sites distant from the parasite. In a previous study, Bozas *et al.* (33) isolated a fraction from adult *F. hepatica* containing a KT inhibitor with activity against trypsin. Furthermore, an *F. hepatica* KT inhibitor was detected among a preparation that was isolated by affinity purification of adult *F. hepatica* excretory-secretory products using anti-cysteine protease monoclonal antibodies (MM3) (49). These studies show that the mature parasite also expresses an FhKT and suggest that the parasite produces more than one KT inhibitor that is possibly temporally regulated alongside the secreted cathepsin Ls. It is noteworthy that the parasite does not secrete any serine proteases during its development in the mammalian host and, therefore, would not require a specific serine-protease inhibitor.

Cysteine protease inhibitors (*e.g.* cystatins) have also been implicated in the modulation of host immune responses (50, 51). We have shown that FhKT1 inhibits human cathepsins L and K, both of which are important proteases of the host immune system. Human cathepsin L functions in the proteolytic processing of antigens in the lysosome for presentation on the surface of antigen-presenting cells (52–54). If taken up by phagocytic cells, FhKT1 inhibition of human cathepsin L could impair this processing and thus dampen adaptive cellular and antibody responses to the parasite. Indeed, previous work in our laboratory has shown that a similarly sized molecule (6 kDa) termed helminth defense molecule secreted by *F. hepatica* is readily taken up by macrophages and impairs antigen processing and presentation (55). Human cathepsin K also plays a critical immune regulatory function as it is involved in Toll-like receptor 9 signaling in dendritic cells (56), and therefore its inhibition would significantly downplay dendritic cell activation. Interestingly, Falcón *et al.* (57) recently reported that a fraction isolated from adult *F. hepatica* that prevented dendritic cell activation in mice contained a KT protease inhibitor, leading the authors to propose that the inhibitor may have an important role in the immune evasion mechanisms of the parasite. Given the potential role of FhKT1 in parasite infection, through the regulation of the major parasite cysteine proteases and cysteine proteases implicated in the host immune system, FhKT1 represents a novel vaccine and drug target.

In summary, the juvenile infective stage of the helminth *F. hepatica* secretes a KT protease inhibitor that atypically lacks the ability to inhibit serine proteases while simultaneously possessing a unique capacity to inhibit cathepsin L-like cysteine proteases. The particular arrangement of functional amino acids predicted to confer this molecule with its unique properties has provided valuable new information and understanding of protease inhibition by KT protease inhibitors. Furthermore, it has opened up the possibility of designing new inhibitors that could target proteases of various mechanistic classes that are important in infectious and non-infectious diseases.

## Experimental Procedures

### 

#### 

##### fhkt1 Gene and Protein Expression in F. hepatica Newly Excysted Juveniles

RNA sequencing and gene expression analysis were carried out in duplicate for 3 and 24 h post-excystment of NEJs as described by Cwiklinski *et al.* (6). The *fhkt1* gene was identified by putative annotation of the *F. hepatica* gene models using *in silico* tools (Uniprot, Gene Ontology (GO), and InterProScan) (6) and BLAST analysis against the *F. hepatica* genome using a previously identified adult *F. hepatica* Kunitz sequence (Fh_Contig2704, Young *et al.* (10)). Differential *fhkt1* gene expression at the 3- and 24-h NEJ life stages was investigated using transcriptome data (European Nucleotide Archive accession number PRJEB6687; Cwiklinski *et al.* (6)) mapped to the gene models identified within the genome (represented as transcripts per kilobase million).

NEJs (North American isolate; Baldwin Aquatics Inc.) were cultured in RPMI 1640 medium for 24 h to obtain their secretory products as described previously (5). These were concentrated by ultrafiltration to 1 mg/ml and stored at −80 °C. Approximately 5 μg of protein was subjected to LDS-PAGE and Western blotting (5) and probed with anti-FhKT1 (1:1000 dilution) raised in mice against the peptide sequence Cys-Glu-Gly-Asn-Asp-Asn-Arg-Phe-Asp-Ser-Lys-Ser-Ser-Cys. As a negative control, another sample was probed with pre-immune mouse antiserum at the same dilution.

NEJ-secreted products were characterized using the proteomics platform of the Quebec Genomics Center, Quebec City, Quebec, Canada. Samples of RPMI 1640 medium in which NEJs (North American isolate; Baldwin Aquatics Inc.) were cultured for 3 and 24 h were analyzed by LC/MS-MS, performed on a TripleTOF 5600 mass spectrometer fitted with a nanospray III ion source (ABSciex Concord, Ontario, Canada), and coupled to an Agilent 1200 HPLC. FhKT1 was identified in MS/MS samples (three separate samples for both time points) analyzed using Mascot version 2.4.0 (Matrix Science) set to search a database composed of gene models identified within the *F. hepatica* genome (version 1.0, 101,780 entries; Cwiklinski *et al.* (6)) and validated using Scaffold (version 4.3.2) as per Cwiklinski *et al.* (7).

##### Immunolocalization of FhKT1 in Newly Excysted Juveniles by Confocal Microscopy

*F. hepatica* metacercariae (Italian isolate; Ridgeway Research, UK) were excysted as described (5), and NEJs were cultured in RPMI 1640 medium (ThermoFisher Scientific) for 3 and 24 h, respectively. The parasites were fixed with 4% paraformaldehyde in 0.1 m PBS (Sigma) overnight at 4 °C and then washed three times with antibody diluent (AbD: 0.1 m PBS containing 0.1% (v/v) Triton X-100, 0.1% (w/v) bovine serum albumin, and 0.1% (w/v) sodium azide). NEJs were then incubated in 0.1 m PBS containing anti-FhKT1 antiserum (prepared in rabbit against the recombinant FhKT1) at a 1:500 dilution, overnight at 4 °C, followed by three washes in AbD. As a negative control, separate samples of 3- and 24-h NEJs were incubated in 0.1 m PBS containing rabbit pre-immune antiserum at a 1:500 dilution. Following washing, all NEJ samples were incubated in a 1:200 dilution of the secondary antibody, fluorescein isothiocyanate (FITC)-labeled goat anti-rabbit IgG (Sigma), overnight at 4 °C, followed by three washes in AbD. To counter-stain muscle tissues, NEJs were incubated in AbD containing 200 μg/ml phalloidin-TRITC overnight at 4 °C. Following three final washes in AbD, NEJs were whole-mounted in a 9:1 glycerol solution containing 0.1 m propyl gallate and viewed using confocal scanning laser microscopy (Leica TCS SP5) under the HCX PL APO CS ×100 oil objective lens. Leica type F immersion oil was used in viewing, and all images were taken at room temperature.

##### Expression of Recombinant FhKT1 and FhKT1Leu^15^/Arg^15^ in the Methylotrophic Yeast P. pastoris

The wild-type *Fhepatica* KT protease inhibitor gene identified by transcriptomic analysis (Fh_Contig2704, Young *et al.* (10); *fhkt1*, Cwiklinski *et al.* (6)) was codon-optimized for expression in *P. pastoris* and synthesized in the puc57 vector (GenScript). A variant of this gene (*fhkt1leu*^15^/*arg*^15^), whereby the P1 leucine residue at position 15 was substituted to an arginine residue (see Fig. 2) was similarly synthesized and codon-optimized for expression in *P. pastoris*. Both synthesized genes did not include the hypothetical N-terminal signal peptide (based on SignalP data). A C-terminal His tag was incorporated into each gene, as well as an N-terminal KpnI restriction site and a C-terminal MlyI restriction site for cloning into the yeast expression vector, Both *fhkt1* and *fhkt1leu*^15^/*arg*^15^ were transformed into One Shot® TOP10 chemically Competent *Escherichia coli* cells (ThermoFisher Scientific) for propagation and ligated into the *pPink*α-*HC* vector before transformation into PichiaPink^TM^ Strain 1 cells (ThermoFisher Scientific) by electroporation. (1500 V for 5 ms), following the manufacturer's protocol. Transformed cells were streaked onto *Pichia* adenine dropout plates and selected for protein expression, as previously described (58).

*fhkt1* and *fhkt1leu*^15^/*arg*^15^-transformed yeast cells were grown in 1 liter of buffered glycerol-complex medium at 28 °C to an *A*_600_ of 20 and then centrifuged at 3000 × *g* for 5 min at room temperature. Pellets were resuspended in 200 ml of buffered methanol-complex medium, and protein expression was induced at 16 °C for a further 3 days by daily addition of 2% methanol. Cells were then pelleted by centrifuging at 3000 × *g,* and the supernatant was collected. The supernatant was diluted 1:4 with column buffer (sodium phosphate buffer, pH 8, 10 mm imidazole) and passed over a column containing a 500-μl bed volume of Ni-NTA beads (Qiagen) at a rate of 0.5 ml/min. The column was washed with 10 ml of wash buffer (50 mm sodium phosphate buffer, pH 8, 20 mm imidazole), and bound recombinant protein was eluted from the column using 5 ml of elution buffer (50 mm sodium phosphate buffer, pH 7, 250 mm imidazole). The eluted protein was buffer-exchanged by dialysis (with a 3-kDa molecular mass cutoff) into PBS over 24 h at 4 °C, aliquoted, and stored at −80 °C until needed.

A Bradford assay was carried out to determine protein yield, and protein purity was visualized by gel electrophoresis on precast NuPAGE Novex 4–12% BisTris protein gels (ThermoFisher Scientific). For Western blot analysis, proteins were transferred to a nitrocellulose membrane and blocked with blocking solution (5% semi-skimmed milk powder in TBST buffer). Blots were probed with the primary antibody, anti-histidine tag (His_6_) monoclonal antibody (ThermoFisher Scientific), which had been diluted 1:10,000 in 1% blocking solution. Blots were then washed three times with TBST (15 min for each wash) before the secondary antibody, alkaline phosphatase-conjugated goat anti-mouse IgG (Sigma) diluted 1:15,000 in TBST buffer, was applied. Antibodies were left to incubate with the membrane for 1 h before being washed three times with TBST. Bound antibody was visualized with the substrate SIGMA FAST^TM^ 5-bromo-4-chloro-3-indolyl phosphate/nitro blue tetrazolium) (Sigma) and imaged using a G:BOX Chemi XRQ imager (Syngene).

##### Inhibition Assays to Determine rFhKT1 and rFhKT1Leu^15^/Arg^15^ Specificity

The inhibitory specificity of rFhKT1 and rFKT1Leu^15^/Arg^15^ was screened using a panel of mammalian serine and cysteine proteases and *F. hepatica* cysteine proteases. Enzymes screened for inhibition included the following: bovine trypsin (Sigma); bovine chymotrypsin (Sigma); human neutrophil elastase (Elastin Products Co.); bovine thrombin (Sigma); porcine pancreatic kallikrein (Sigma); human cathepsin G (Elastin Products Co.); human cathepsin B (Sigma); human cathepsin K (Enzo Life Sciences); human cathepsin L (Sigma); human cathepsin S (Sigma). Purified *F. hepatica* cathepsin L1 (FhCL1), *F. hepatica* cathepsin L2 (FhCL2), *F. hepatica* cathepsin B1 (FhCB1), and *F. hepatica* cathepsin B2 (FhCB2) were expressed as functionally active recombinant forms in *P. pastoris* in our laboratory (36).

Reaction conditions and substrates employed for measuring the activity of each protease are presented in Table 4. rFhKT1 and rFhKT1Leu^15^/Arg^15^ (300 nm) were first incubated with each protease in a 100-μl volume reaction buffer for 15 min at 37 °C. Reaction volumes were then brought to 200 μl with the addition of fluorogenic substrate dissolved in reaction buffer, and the proteolytic activity was measured as RfU in a PolarStar Omega spectrophotometer (BMG LabTech, UK). Enzyme inhibition studies were performed with FhCL1 and FhCL2 at different pH values ranging from 4.5 to 8.5 using citrate phosphate buffer. All assays were carried out in triplicate.

**TABLE 4:** **Fluorogenic assay conditions for each protease that inhibition was screened against**

Enzyme	Assay Buffer	Substrate
*F. hepatica* cathepsin L1 (2.7 nm)	50 mm citrate phosphate buffer, pH 5.5, 2 mm dithiothreitol, 0.01% Brij L23	Z-Leu-Arg-NHMec (20 μm)
*F. hepatica* cathepsin L2 (5 nm)	50 mm citrate phosphate buffer pH 5.5, 2 mm DTT, 0.01% Brij L23	Z-Leu-Arg-NHMec (20 μm)
*F. hepatica* cathepsin B1 (34 nm)	50 mm citrate phosphate buffer pH 5.5, 2 mm DTT, 0.01% Brij L23	Z-Phe-Arg-NHMec (20 μm)
*F. hepatica* cathepsin B2 (180 nm)	50 mm citrate phosphate buffer pH 5.5, 2 mm DTT, 0.01% Brij L23	Z−Gly−Pro−Arg−NHMec (20 μm)
Human cathepsin L (0.2 nm)	50 mm citrate phosphate buffer pH 5.5, 2 mm DTT, 0.01% Brij L23	Z-Phe-Arg-NHMec (20 μm)
Human cathepsin K (2 nm)	50 mm citrate phosphate buffer pH 5.5, 2 mm DTT, 0.01% Brij L23	Z-Phe-Arg-NHMec (20 μm)
Human cathepsin S (2 nm)	100 mm sodium acetate buffer, pH 5.6, 1 mm EDTA, 1 mm DTT, 0.01% Brij L23	Z-Val-Val-Arg-NHMec (20 μm)
Human cathepsin B (3.6 nm)	50 mm citrate phosphate buffer pH 5.5, 2 mm DTT, 0.01% Brij L23	Z-Phe-Arg-NHMec (20 μm)
Trypsin (42 nm)	100 mm Sodium Phosphate Buffer pH 7.6, 1 mm HCl, 20 mm Ca, 0.01% Brij L23	Z-Leu-Arg-NHMec (20 μm)
Chymotrypsin (40 nm)	100 mm sodium phosphate buffer, pH 7.6, 1 mm HCl, 20 mm calcium, 0.01% Brij L23	Suc-Ala-Ala-Pro-Phe-NHMec (20 μm)
Neutrophil elastase (50 nm)	100 mm Hepes, 500 mm NaCl, pH 7.5, 0.01% Brij L23	MeOSuc-Ala-Ala-Pro-Val-NHMec (20 μm)
cathepsin G (100 nm)	100 mm Tris pH 7.5, 0.01% Brij L23	Suc-Ala-Ala-Pro-Pro-NHMec (100 μm)
Thrombin (43 pm)	100 mm Tris, 150 mm NaCl, pH 7.4, 0.01% Brij L23	Z-Gly-Pro-Arg-NHMec (2 μm)
Kallikrein (150 nm)	20 mm Tris-HCl, pH 7.8, 100 mm NaCl, 0.01% Brij L23	Z-Phe-Arg-NHMec (20 μm)

To determine inhibition constants, decreasing concentrations (nm) of rFhKT1 and rFhKT1Leu^15^/Arg^15^ were exposed to each protease by serial dilution. Substrate concentrations were equal to the known *K_m_* values of the cysteine proteases. *K_i_* values were determined using the Morrison equation for tight-binding inhibition (built into GraphPad Prism 5.0). Briefly, initial velocities were fitted to Morrison's equation (Equation 1), and the resulting apparent *K_i_* (*K*_*i*_^app^) was fitted to Equation 2 to determine the *K_i_* for FhKT1 and FhKT1Leu^15^/Arg^15^ with each cysteine protease inhibited (59).

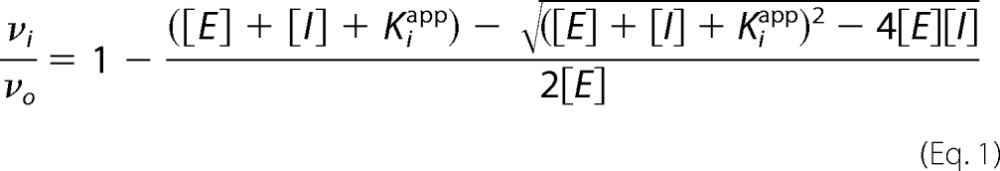


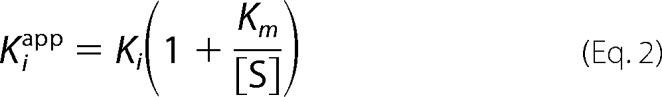
 υ*_i_* is the initial velocity of a reaction containing the inhibitor, and υ_0_ is the initial velocity of a reaction without the inhibitor; [*E*] is the concentration of enzyme catalytic sites; [*I*] is the inhibitor concentration; [S] is the substrate concentration, and *K_m_* is the known Michaelis-Menten constant for a given substrate with a given enzyme. Inhibitor concentrations ranged from 20 to 0.1 nm, providing a range of concentrations above and below the enzyme concentrations used for the cysteine proteases inhibited (see Table 1).

To determine the importance of the tertiary structure for the inhibitory activity of rFhKT1 and rFhKT1Leu^15^/Arg^15^, samples of both inhibitors were reduced and alkylated using tributylphosphine (Sigma) and acrylamide (Sigma), and their inhibition of FhCL1 and trypsin was measured. Briefly, the inhibitors were diluted in 40 mm Tris, 5 mm TBP, and 10 mm acrylamide and incubated for 2 h at room temperature before quenching the alkylation process by addition of 10 mm DTT (Sigma). For inhibition assays, untreated and reduced/alkylated rFhKT1 and rFhKT1Leu^15^/Arg^15^ were added to the fluorogenic assays (performed as described above) at a final concentration of 25 nm (sufficient for >95% protease inhibition by the non-treated inhibitors). Samples of rFhKT1 and rFhKT1Leu^15^/Arg^15^ taken after quenching of the alkylating reagent samples were kept at 4 °C for 10 days and then assayed for inhibitory activity as above.

##### Inhibition of rFhCL1 and rFhCL2 Auto-catalytic Activation by rFhKT1 and rFhKT1Leu^15^/Arg^15^

Unprocessed FhCL1 zymogen (∼37 kDa, 1.35 μm, ∼1 μg) and rFhKT1 or rFhKT1Leu^15^/Arg^15^ (6.5 μm, ∼1 μg) were premixed in citrate phosphate buffer, pH 4.5, and incubated at 37 °C for 45 min. For comparison, FhCL1 zymogen was also incubated in citrate phosphate buffer, pH 4.5, in the presence of 1 μg of BPTI. As a control for auto-catalytic activation of FhCL1, activation was also carried out in the absence of inhibitor, which has previously been shown to be successful at pH 4.5 (34). Following incubation at 37 °C for 45 min, the cysteine protease inhibitor E-64 (100 μm) was immediately added to the samples to prevent any further potential proteolysis by the cysteine protease. A sample of the FhCL1 zymogen was also used for comparison, whereby E-64 was immediately added to prevent any auto-catalytic activation. Samples were analyzed by LDS-electrophoresis using precast NuPAGE Novex 4–12% BisTris protein gels (ThermoFisher Scientific), stained with Biosafe Coomassie (Bio-Rad), and imaged using a G:BOX Chemi XRQ imager (Syngene).

##### Stability of the rFhKT1-Cysteine Protease Complex

*F. hepatica* cathepsin L1 that was fully activated at pH 4.5 for 1 h (25 kDa, 2 μm, ∼1 μg) was incubated in the presence or absence of rFhKT1 (6.5 μm, ∼1 μg) in citrate phosphate buffer, pH 5.5, and incubated at 37 °C. The reaction was monitored for 6 h using the peptide substrate Z-Phe-Arg-NHMec. Samples of the reaction were removed at 1, 2, and 6 h and added immediately to the cysteine protease inhibitor E-64 (100 μm) to prevent any further potential proteolysis by the cysteine protease. These samples were then analyzed by LDS-electrophoresis and by Western blot analysis with rabbit anti-rFhKT1 polyclonal antibodies (prepared by Eurogentec, Liège, Belgium) and anti-His tag monoclonal antibodies (Sigma). To determine the long term stability of FhCL1 inhibition by rFhKT1, these were mixed as described above, and enzyme activity in samples with and without inhibitor was monitored over the course of 7 days.

##### Pulldown of Cysteine Protease from Adult F. hepatica Excretory-Secretory Products

Adult *F. hepatica* parasites (flukes) were obtained from lamb livers sourced from an abattoir in Northern Ireland, United Kingdom. Upon collection, adult *F. hepatica* were washed with PBS (containing 0.1% glucose) and maintained in RPMI 1640 culture medium (ThermoFisher Scientific) containing 2 mm l-glutamine (ThermoFisher Scientific), 30 mm Hepes (ThermoFisher Scientific), and 0.1% glucose at two flukes/ml. Ten μg of rFhKT1 was added to one culture of media, although, as a control, no inhibitor was added to a similar parasite culture. After 5 h of incubation at 37 °C, the parasite culture media were collected and centrifuged at 300 × *g* for 10 min and then at 700 × *g* for 30 min (both at 4 °C) to remove large debris (*e.g.* parasite eggs). Then, a 500-μl bed volume of pre-washed Ni-NTA beads was added to each media and mixed by rotating for 1 h at 4 °C. The Ni-NTA beads were then centrifuged in a bench top microcentrifuge at 100 × *g,* and the supernatant was decanted and stored. The pelleted Ni-NTA beads were washed twice with wash buffer before elution with 100 μl of elution buffer (see above). Column flow-through and eluted samples were analyzed on precast NuPAGE Novex 4–12% BisTris protein gels (ThermoFisher Scientific), stained with Biosafe Coomassie, and imaged using a G:BOX Chemi XRQ imager (Syngene).

The cysteine protease activity of the media before and after the pulldown with Ni-NTA beads was determined by fluorogenic assays using the cathepsin L substrate Z-Phe-Arg-NHMec. An ∼26-kDa protein band that had been pulled down from the medium to which rFhKT1 was added was characterized by LC-MS/MS analysis at the Fingerprints Proteomics Facility, University of Dundee, Scotland, United Kingdom.

##### Molecular Modeling of rFhKT1 and rFhKT1Leu^15^/Arg^15^ Protease Inhibition

The crystal structure of BPTI with the PDB code of 3OTJ (60) was used to build the homology model of FhKT1 using Prime 4.0 (61, 62) with the energy-based setting. The obtained homology model was first docked to the crystal structure of FhCL1 previously reported by us (PDB code 2O6X) (36) using the HEX 6.1 protein-protein docking program (63) and subjected to molecular dynamics simulations using MacroModel 10.8. In docking, the shape-based and electrostatic-based assessment of binding was conducted with over 100 generated and analyzed docking solutions. The docking solution in which the P1 Leu^15^ of FhKT1 is pointed to the active site of FhCL1 was selected for molecular dynamics simulations. 10 ns of molecular dynamics simulations and 5000 steps of followed minimization were conducted in implicit solvent at a temperature of 300 K to obtain the final complex. OPLS_2005 force field was used in MacroModel calculations. A similar docking and simulation procedure was used to study FhKT1 binding with human cathepsins K, L, S, and B (PDB codes 1YK7, 4AXM, 3N3G, and 3K9M, respectively). The image with the molecular models was prepared with Maestro 10.2.

## Author Contributions

D. S. conceived the ideas, conducted most of the experiments, analyzed the results, and was involved in experimental design and wrote the paper. I. G. T. performed the molecular modeling and docking simulations and was involved in writing and editing the manuscript. H. L. J. performed the immunolocalization studies. O. D. performed enzymatic assays and characterizations. M. W. R. and J. D. were involved in experimental design, provided materials, and were involved in the writing and editing of the manuscript. K. C. was involved in sequence analysis and interpretation of results and was involved in writing and editing the manuscript. J. P. D. conceived the ideas, contributed to data analysis, was involved in writing the manuscript, was the principle investigator, was involved in designing the study, and provided the majority of the resources utilized in the study.

## Supplementary Material

Supplemental Data

## Footnotes

5 The abbreviations used are:

NEJ: newly excysted juvenile
KT: Kunitz-type
E-64: *trans*-epoxysuccinyl-l-leucylamido(4-guanidino)butane
NHMec: 7-amino-4-methylcoumarin
Ni-NTA: nickel-nitrilotriacetic acid
BPTI: bovine pancreatic trypsin inhibitor
RfU: relative fluorescent unit
PDB: Protein Data Bank
RPMI 1640 medium: Roswell Park Memorial Institute 1640 medium
LDS-PAGE: lithium dodecyl sulfate-PAGE
AbD: antibody diluent
TRITC: tetramethylrhodamine isothiocyanate
BisTris: 2-[bis(2-hydroxyethyl)amino]-2-(hydroxymethyl)propane-1,3-diol
Z: benzyloxycarbonyl.
