# Supplementary material for: Unexpected Activity of a Novel Kunitz-type Inhibitor: INHIBITION OF CYSTEINE PROTEASES BUT NOT SERINE PROTEASES
Source: J Biol Chem. 2016 Jul 15;291(37):19220–34. doi: 10.1074/jbc.M116.724344 (PMC5016662; doi:10.1074/jbc.M116.724344)
Supplement: Supplemental Data [file supp_291_37_19220__index.html]

Unexpected Activity of a Novel Kunitz-Type Inhibtior: Inhibition of Cysteine Proteases but not Serine Proteases — Unexpected Activity of a Novel Kunitz-Type Inhibitor: Inhibition of Cysteine Proteases but not Serine Proteases — Unexpected Activity of a Novel Kunitz-type Inhibitor — Kunitz-type Inhibitor with a Unique Inhibition Profile — Supplemental Data 

# Unexpected Activity of a Novel Kunitz-type Inhibitor

## Supplemental Data

- Supplemental File 1 (.xlsx, 13 KB) - Supplemental File 1: Peptide data identifying FhKT1 in Fasciola hepatica newly excysted juveniles
- Supplemental File 2 (.xls, 38 KB) - Supplemental File 2: Peptide data identifying proteins from FhKT1 pull-down of adult Fasciola hepatica ES
